# Correlation between Arterial Lactate and Central Venous Lactate in Children with Sepsis

**DOI:** 10.1155/2016/7839739

**Published:** 2016-10-16

**Authors:** Jaime Fernández Sarmiento, Paula Araque, María Yepes, Hernando Mulett, Ximena Tovar, Fabio Rodriguez

**Affiliations:** ^1^Pediatric Critical Care Department, Fundación Cardioinfantil, Universidad de la Sabana, Bogotá, Colombia; ^2^Pediatric Critical Care Department, Universidad del Rosario, Bogotá, Colombia; ^3^Pediatrics and Research Department, Universidad de la Sabana, Bogotá, Colombia

## Abstract

*Introduction*. Lactate is an important indicator of tissue perfusion. The objective of this study is to evaluate if there are significant differences between the arterial and central venous measurement of lactate in pediatric patients with sepsis and/or septic shock.* Methods*. Longitudinal retrospective observational study. Forty-two patients were included between the age of 1 month and 17 years, with a diagnosis of sepsis and septic shock, who were admitted to the intensive care unit of a university referral hospital. The lactate value obtained from an arterial blood sample and a central venous blood sample drawn simultaneously, and within 24 hours of admission to the unit, was recorded.* Results*. The median age was 2.3 years (RIC 0,3–15), with a predominance of males (71.4%), having a 2.5 : 1 ratio to females. Most of the patients had septic shock (78.5%) of pulmonary origin (50.0%), followed by those of gastrointestinal origin (26.1%). Using Spearman's Rho, a 0.872 (*p* < 0.001) correlation was found between arterial and venous lactate, which did not vary when adjusted for age (*p* < 0.05) and the use of vasoactive drugs (*p* < 0.05).* Conclusion*. There is a good correlation between arterial and venous lactate in pediatric patients with sepsis and septic shock, which is not affected by demographic variables or type of vasoactive support.

## 1. Introduction

Sepsis is a disease characterized by a hypercatabolic state, with an increased tissue oxygen demand. When there is an imbalance between the supply and demand of oxygen, both tissue hypoperfusion and hypoxia lead to anaerobic metabolism with the ultimate production of lactate [1].

Under critical conditions homeostasis is altered, producing reversible or irreversible multiple organ injury. The length of exposure to the damaging stimulus determines the reversibility of the organ dysfunction (2-3). Therefore, early detection of sepsis and organ dysfunction is important in critically ill patients in order to begin appropriate therapeutic measures [1, 2].

Traditionally, identification of organ dysfunction in patients was made based on clinical signs and symptoms (oliguria, altered consciousness) and identification of circulatory failure and consequent tissue hypoperfusion, with the presence of persistent and refractory hypotension (4-5). This approach is complicated due to the lack of sensitivity of the clinical findings to predict the presence or absence of an organ lesion or tissue hypoperfusion, especially in the pediatric population with physiological variables changing and complementary tests are typically needed [1].

Serum lactate is a biomarker which estimates the probability and extent of tissue hypoperfusion and also evaluates the therapeutic response and prognosis [2]. A value greater than 4 mmol/L in severe sepsis indicates the need for aggressive resuscitation according to the sepsis consensus [3]. Arterial lactate levels are the ideal sample for determining hyperlactatemia, with the latter being a severity marker [3] which may be more specific than the customary hemodynamic measurements and therefore must be interpreted according to the patient's context.

In light of the difficulty in drawing an arterial sample in patients, especially in the pediatric age group, the need to employ another, more accessible, type of sample for its measurement is proposed, in order to determine this pathological condition. For this reason, a correlation between arterial and venous lactate in children with sepsis and/or septic shock is sought; considering that there are no existing studies in the pediatric population the objective of our study was to assess whether there was a correlation between arterial and venous lactate in children with sepsis [4, 5].

## 2. Materials and Methods

### 2.1. Study Design

A longitudinal retrospective observational study diagnostic test was carried out on Pediatric Intensive Care Unit patients at the Fundación Cardioinfantil-Instituto de Cardiología, a quaternary care institution in Bogotá, Colombia, South America, where there is electronic chart review. The study was previously analyzed and approved by this institution's Ethics Committee and all requirements were met including informed consent.

### 2.2. Study Population

**Figure 1:**
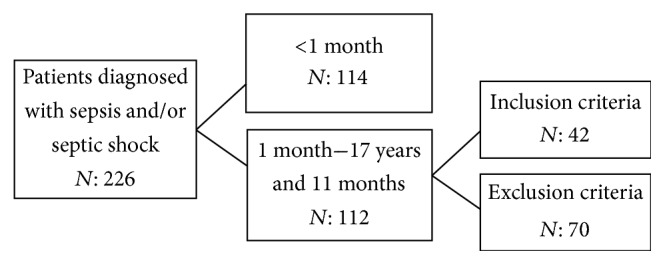
Flow chart of children included in the study.

Patients admitted to the Pediatric Intensive Care Unit of the Fundación Cardioinfantil between January 1, 2009, and May 31, 2012, with a diagnosis of sepsis and/or septic shock, according to the 2005 International Pediatric Sepsis Consensus were included [3]. Patients from 1 month old to 17 years and 11 months old who were admitted to the Pediatric Intensive Care Unit with a diagnosis of sepsis and/or septic shock, who required invasive or noninvasive mechanical ventilation, and who had central venous access as well as an arterial line were included. Children with congenital cardiopathies, intra- or extracardiac shunting, diabetic ketoacidosis, insulin use at the time of blood sampling or 24 hours priorly, suspected inborn errors of metabolism, or patients with intoxication, chronic or acute liver failure, and chronic renal failure with or without dialysis, were not included [4] (Figure 1).

The electronic medical records were reviewed, and the required information was recorded on a questionnaire developed for this purpose. The recorded arterial and central venous gas samples corresponded to the first sample taken simultaneously within the first 24 hours following admission as is usual protocol in the Pediatric Intensive Care Unit. The information recorded in the medical records was verified with the institution's clinical laboratory's official data base. The blood drawing technique was reviewed with the PICU nursing staff; the technique is standardized for drawing arterial and venous blood samples simultaneously by two staff members. The arterial blood was obtained from a radial or femoral arterial line, while the venous blood was extracted through a central venous catheter located in the subclavian, internal jugular vein (the distal tip should lie in the superior vena cava in the cavoatrial union) or femoral vein. All samples were collected in heparinized syringes, leaving no spaces or bubbles in them. For the arterial blood draw, 5 mL of blood was extracted through the arterial line, and then 1 mL was collected in the heparinized syringe; following this, the initially extracted 5 mL was returned through the arterial line, thus avoiding the anemization of patients. For the venous sample, 10 mL of blood was extracted through the central venous catheter, and then 1 mL was collected in the heparinized syringe, and the original 10 mL of blood was returned to the patient. The samples were immediately sent through the pneumatic tube system, guaranteeing their processing in the least amount of time possible, not greater than 5 minutes by institutional standards. All blood samples were analyzed on the Siemens Rapidlab® 1265 series blood gas machine, which performed an analysis of arterial and central venous gases, reporting the lactate levels of each sample type. The time elapsed between blood samples draws and processing in the laboratory was a maximum of 15 minutes as part of a standardized protocol at our institution. A data base was created with the variables of interest, and statistical analysis was performed.

### 2.3. Statistical Analysis

All the information was collected in a database whose source was the electronic medical record. The SPSS version 15.0® program was used for data analysis. A descriptive analysis of the variables was carried out, using means, medians, and standard deviation (SD) for continuous variables and proportions in categorical variables. A comparison of the means, medians, and SD of the lactate from the arterial and central venous samples was performed using Mann–Whitney* U* test. The normality of the arterial and central venous lactate was evaluated with the Kolmogorov-Smirnov test, finding that the lactates did not fulfill the assumption of normal distribution [6]. Therefore, Spearman's correlation coefficient was then calculated and a dispersion diagram was obtained to determine the basic nature of the relationship between the variables of arterial lactate, central venous lactate, weight, and age.

## 3. Results

### 3.1. Descriptive Analysis

The sample size was 42 patients, with median age of 4.5 years (RIC 0,3–15,4). There were 12 females (28.5%) and 30 males (71.4%), with a male to female ratio of 2.5 : 1. The main diagnosis found was septic shock in 78.6% of cases (*n* = 33) and 21,4% was sepsis (*n* = 9); the main source was pulmonary origin, 50%, followed by gastrointestinal, 26.1% (mainly gastroenteritis) (Table 1). There was a median 1.4 mmol/L (SD 0.88 mmol/L) lactate measurement for arterial lactate, observing a minimum value of 0.3 and a maximum of 4.8 mmol/L. For central venous lactate, the median was 1.6 mmol/L (SD 0.9 mmol/L), with a minimum of 0.2 mmol/L and a maximum of 4.7 mmol/L.

### 3.2. Bivariate Analysis

**Figure 2:**
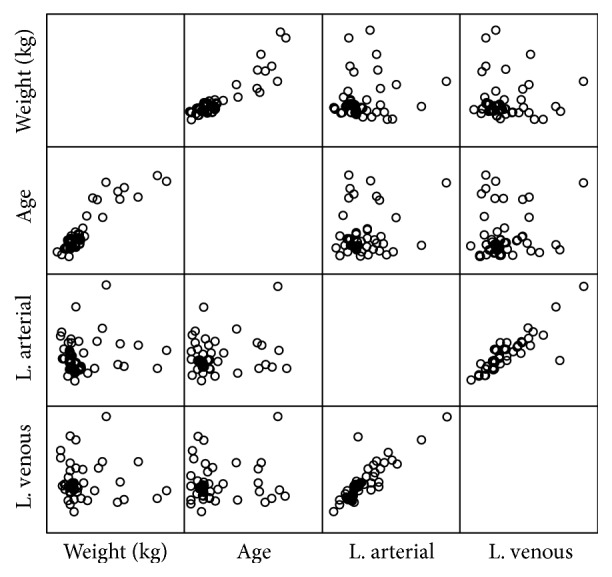
Dispersion diagram of the study variables.

A bivariate analysis was carried out by calculating the correlation coefficient using Spearman's Rho statistical test. The correlation for central venous and arterial lactate proved to be statistically significant (*p* < 0.001), with Spearman's index of 0.897. There was no correlation between arterial lactate and venous lactate with the weight and age variables (Figure 2).

Likewise, the relationship was analyzed between the use of vasoactive drugs (epinephrine, dopamine, and norepinephrine) during hospitalization and potentially increase lactate values with the different numerical variables, without finding any difference (*p* = 0.53) (Table 2).

In bivariate analysis between the different numerical variables and categorical variables, through the medium compared with the Mann–Whitney* U* test, the behavior of the blood lactate and central venous according to gender, type of sepsis, and vasoactive drugs was determined (Table 3).

### 3.3. Linear Regression

The analysis was completed with a nonparametric quantile regression model with the median. Application of the regression model showed a beta level between arterial lactate and central venous lactate of 0.917. The confounding variables were proven not to influence the beta values (0.92) (Table 4).

## 4. Discussion

Our study found a good correlation between arterial and central venous lactate in pediatric patients with sepsis and/or septic shock who are in intensive care (Spearman's index 0.897 and *p* < 0.001). When we analyzed the results we found no statistically significant differences between arterial lactate and central venous lactate with respect to age, weight, and diagnosis, keeping in mind that the severity and stages of illness can vary rapidly [3, 7, 8]. Likewise, in spite of the fact that body surface changes with age (*p* = 0.14) and weight (*p* = 0.74), significant differences were not found either, which makes lactate a reliable indicator, not influenced by these variables [1, 5, 9]. Furthermore, it was noted that the use of vasoactive drugs and medications described in the literature as hyperlactatemia producers did not, in our sample, alter the central venous lactate levels with respect to the arterial lactate (*p* = 0.068). This finding is very important in critically ill patients in whom it is sometimes difficult to obtain arterial access due to their state of shock or the high vasopressor support, which makes the procedure difficult. In addition, the consideration that arterial and venous lactate results are correlated, regardless of how compromised their removal mechanism may be (due to lactate dehydrogenase or to the oxidative capacity of the tissues), has a significant added value because it provides the clinician with more tools with which to evaluate the critical patient when an arterial or central venous line is not available. In other words, regardless of the origin of lactate analysis both production and the arterial and venous elimination behave uniformly.

No pediatric studies were found in the reviewed literature which would allow a comparison of the central venous and arterial lactate results in septic patients with our findings. Our study suggests conclusions which have been concordant with findings obtained in adult patients, in whom few studies have been carried out on this particular aspect. Bucuvalas et al. [9] compared the arterial lactate and peripheral venous lactate values of patients over 18 years old with or without sepsis who presented to the emergency room, finding a good correlation of 0.89 (*p* < 0.05) between these values. More recently, Nascente et al. [1] evaluated the correlation between the lactate values obtained from different anatomic sites in a population of adult patients with severe sepsis or septic shock. This study showed that central venous blood lactate has a good correlation with arterial lactate levels, with a correlation index of 0.84 (*p* < 0.00001).

One of the limitations of our study is that simultaneous measurement of arterial and central venous lactate was not always performed. In addition, in spite of the fact that the sample size was calculated according to the findings in previous studies, it corresponds to the experience of a single reference site, which should be corroborated with prospective studies in the pediatric population which would also determine the sensitivity and specificity of these tests as clinical determinants in patients being treated for sepsis or septic shock.

Within the reviewed literature, our study is the first research to compare arterial and central venous lactate in critically ill children with a potentially lethal pathology such as sepsis and septic shock. Likewise, although lactate is analyzed at a specific point in time, having central venous lactate as a reliable alternative to arterial lactate provides a monitoring tool and, above all, can serve as another tool to help determine the severity of the illness. A goal-guided approach, according to the studies by Nguyen et al. [10], has shown that it modifies the course of the disease and may reduce mortality.

Technical difficulties in obtaining an arterial line in pediatrics are very common, especially when the patient is in shock, but also for all those cases where an arterial vascular access is not necessary or during the initial phase of stabilization before an arterial vascular access can be obtained. These are why being able to assume that lactate obtained from a central vein is just as reliable as arterial lactate provides alternatives when establishing therapeutic goals on the admission of our patients.

We recommend carrying out prospective studies that will evaluate the behavior of lactate over time, its clearance, and its relationship to the clinical course of the patient.

## 5. Conclusion

There is a good correlation between arterial and central venous lactate in children with septic shock, after statistically adjusting the different variables inherent to the critically ill patient, such as the use of vasoactive support, medications, and demographic variables, serving as a good indicator of tissue perfusion in critically ill children.

## Figures and Tables

**Table 1:** Demographic variables.

*Age (median years)*	4.5
*Sex*	*n* (%)
Female	12 (28,5)
Male	30 (71,4)
*Weight*	*n* (%)
<10 kg	11 (27)
11–20 kg	21 (50)
21–30 kg	4 (9,5)
>30 kg	6 (14)

**Table 2:** Features of sepsis in the population. The difference between arterial and venous lactate according to the severity of the disease, its origin, and the use of vasopressors described.

Variables	*n* (%)	Arterial versus venous lactate difference—*p* value
*Severity of disease*		0.8
Sepsis	9 (21,4)	
Septic shock	33 (78,5)	
*Etiology of sepsis *		0.1
Pulmonary	21 (50,0)	
Gastrointestinal	11 (26,1)	
*Vasopressors*		0.53
Yes	38 (90,4)	
No	4 (9,5)	

**Table 3:** Bivariate analysis arterial lactate versus venous lactate. Relationship between arterial and venous lactate and factors like sex, vasopressors, severity of illness, and other medications such as antibiotics and sedatives.

	Arterial lactate *n* = 42	*p* value	Venous lactate *n* = 42	*p* value
*Sex*				
Female (mean ± SD)	1.905 (1.128)	0.031	2.188 (1.226)	0.522
Male (mean ± SD)	1.295 (0.725)		1.374 (0.740)	
*Type of sepsis*				
Sepsis (mean ± SD)	1,594 (0,667)	0,624	1,59 (0,758)	0,124
Septic shock (mean ± SD)	1,435 (0,947)		1,612 (1,023)	
*Vasoactive*				
Yes (mean ± SD)	1,452 (0,912)	0,915	1,617 (0,992)	0,068
No (mean ± SD)	1,63 (0,718)		1,51 (0,739)	
*Medications*				
Yes (mean ± SD)	1,545 (1,129)	0,762	1,707 (1,211)	0,641
No (mean ± SD)	1,386 (0,534)		1,497 (0,598)	

**Table 4:** Nonparametric regression model. Model description of relationship between arterial and venous lactate factors that may affect the outcome.

Arterial lactate	Coefficient	Std. err	*t*	*p*	95% CI	
Medications	−0,0803	0,1347	−0,6	0,555	−0,3541	0,1933
Weight	−0,0532	0,1638	−0,33	0,747	−0,3862	0,2797
Vasoactive	0,1257	0,2013	0,62	0,537	−0,2835	0,535
Diagnosis	−0,0439	0,1765	−0,25	0,805	−0,4027	0,3148
Sex	0,1973	0,1673	1,18	0,246	−0,1427	0,5374
Age	0,0529	0,0353	1,5	0,143	−0,0188	0,1246
Venous lactate	0,924	0,0748	12,35	0,000	0,7719	1,076
Constant	−0,3501	0,5095	−0,69	0,497	−1,3856	0,6853

## Additional Points

(1) Lactate is a good coadjuvant for establishing tissue perfusion and guiding fluid resuscitation in pediatric patients with septic shock. (2) The existing correlation between arterial and central venous lactate allows the latter to be taken as a reliable value for making clinical decisions. (3) The comprehensive evaluation of the patient, correlating it with clinical and paraclinical variables, allows the establishment of clear objectives which may modify the course of children with septic shock.
